# Perspectives of health care providers on obstetric point-of-care ultrasound in lower-level health facilities in Kenya

**DOI:** 10.1016/j.midw.2024.104196

**Published:** 2025-01

**Authors:** Lister N. Onsongo, Sarah C. Bett, Grace W. Gachuiri, Stephen N. Njuguna, Jacob W. Masika, George O. Otieno, Anthony K. Wanyoro, Matthew S. Haldeman, Dilys Walker, Nicole Santos, Grace K. Githemo

**Affiliations:** a Community and Reproductive Health Nursing Department, School of Health Sciences- Kenyatta University, Kenya; b Medical-Surgical Nursing & Pre-clinical Sciences, School of Health Sciences-Kenyatta University, Kenya; c Health Management and Informatics, School of Health Sciences-Kenyatta University, Kenya; d Obstetrics and Gynecology Department, School of Health Sciences-Kenyatta University, Kenya; e Global Ultrasound Institute and Department of Family and Preventive Medicine, University of South Carolina School of Medicine, Kenya; f Institute for Global Health Sciences, University of California, San Francisco, United States

**Keywords:** Nurse/Midwives, Maternal health, Antenatal care, Kenya, Implementation, Obstetric Point of Care Ultrasound

## Abstract

**Background:**

Ultrasound technology has become integral in antenatal care for its diagnostic effectiveness and potential to improve maternal and neonatal outcomes. Despite its proven benefits, challenges persist in its widespread adoption, particularly in low-resource settings like Kenya. Aim: The aim of this study was to explore the perspectives of healthcare providers regarding the integration of obstetric point-of-care ultrasound into routine maternal services in low-level facilities Kenya.

**Methods:**

Using a descriptive qualitative study embedded in a large scale implementation study 76 healthcare providers who had undergone obstetric point-ofcare ultrasound training and were providing maternal services were purposively sampled from healthcare facilities across eight counties. Data was collected using structured audiotaped interviews, which were transcribed, and analyzed using thematic analysis.

**Results:**

Five main themes with several subthemes emerged from the analysis: (1) Clinical Decision-Making (2) Quality of Services, (3) Training, (4)Technology Issues, and (5) Sustainability.

**Discussion:**

Findings from this study suggest that use of obstetric Point-of-Care Ultrasound in resource-limited primary care settings, can enhance clinical decision making and influence patient management, ultimately resulting in significant health outcomes.

**Conclusion:**

Equipping health care providers with skills to conduct obstetric point of care ultrasound can lead to better-informed clinical decisions and ultimately contribute to improved health outcomes in underserved populations.

## Highlights

• O-POCUS enables early identification of conditions like of fetal presentation, multiple gestation, and placental issues, leading to timely interventions.
• The findings show the role of O-POCUS in enhancing clinical decision-making and service quality, in lower level facilities in LMICs.
• This paper contributes insights from diverse HCPs, to the understanding of O-POCUS implementation in LMICs, emphasizing the need for comprehensive strategies to improve maternal care.

## Introduction

Ultrasound is an important and effective diagnostic tool in enhancing care among pregnant women. The World Health Organization (WHO) recommends one ultrasound before 24 weeks of gestation to estimate gestational age, detect anomalies, and multiple gestation early in pregnancy (WHO 2016; WHO, 2022). Obstetric point-of-care ultrasound (O-POCUS) is an effective intervention adjunct to bedside clinical evaluation (Wachira et al., 2023) and can improve diagnostic accuracy, time to diagnosis, and procedural safety for pregnant mothers (Mubuuke & Nassanga, 2023). However, data on maternal and neonatal outcomes is inconsistent. While Goldenberg et al. (2018) reported that introduction of routine ultrasound in antenatal care (ANC) did not show an improvement in maternal and neonatal mortality, several studies conducted in low- and middle-income countries (LMICs) reveal that O-POCUS is associated with benefits that include improved access to ultrasonography services, timely diagnosis and referral of high-risk pregnancies, and improved maternal and neonatal outcomes (Hall et al., 2021; Kumar et al., 2023; Wanjiku et al., 2024)

Adoption of O-POCUS in routine obstetrics and midwifery practice in LMICs remains a challenge. Factors such as lack of ultrasound machines, shortage of sonographers, radiographers and other trained staff, lack of standardized O-POCUS training, inadequate resources, and healthcare infrastructure fragmentation hinder O-POCUS implementation (Matiang'i et al., 2021; Mubuuke & Nassanga, 2023; Wachira et al., 2023). To address some of these challenges, task sharing has been shown to strengthen and expand the health workforce and improve access to health services, including O-POCUS (Abrokwa et al., 2022).

In Kenya, O-POCUS is a relatively new technology that has reached few rural settings. Kenya introduced its first O-POCUS training program in 2013 to address a health workforce shortage in undertaking ultrasound scans for underserved populations. However, to date, its objective of increasing access to ultrasound services in rural areas has not been achieved due to limited access to the services and cost of care in primary healthcare settings (Matiang'i et al., 2021). In some cases, pregnant mothers must travel to distant places to receive O-POCUS services and encounter long waiting times, which lowers service utilization and increases the risk of adverse pregnancy outcomes (Hall et al., 2021). Additionally, most healthcare providers have little or no experience with O-POCUS use and most hospitals have trained only a few providers, which affects service utilization and access. Consequently, these barriers lower healthcare providers’ capability, opportunity, and motivation in performing O-POCUS (Wachira et al., 2023).

Since the introduction of O-POCUS use in Kenya, limited data has emerged describing the perspectives of healthcare providers working in lower-level facilities and the integration of O-POCUS into routine care. Kumar et al. (2023) conducted a study that examined acceptability, suitability, and utility of nurse-led O-POCUS with telemedicine at two public hospitals in Nairobi, Kenya, but the study findings are not generalizable to rural settings, where O-POCUS has the greatest potential benefit. Therefore, this qualitative study was conducted to explore the perspectives of healthcare providers in rural lower-level facilities on the integration of O-POCUS into routine ANC services, six months following training and introduction of O-POCUS across eight counties in Kenya.

## Participants, ethics and methods

### Participants

For this qualitative study, participants were purposively sampled from level 2–4 healthcare facilities across eight selected counties. We targeted a minimum sample of 10 healthcare providers per county across different cadres. The targeted large sample size is not typical of qualitative studies, however a key advantage of a large-scale qualitative research approach is the collaborative nature of working in a team (Hunt, Moloney & Fazio, 2011). The inclusion criteria included healthcare providers who completed the O-POCUS training and continued to provide maternal services at the time of data collection.

### Design and data collection methods

This is a qualitative descriptive design embedded in a large scale implementation study across eight Counties. Face to face in-depth interviews lasting between 30 and 60 min were conducted in English using a pre-tested structured interview guide by eight interviewers. Interviews were scheduled through appointments and at the convenience of the healthcare providers. Interviews were conducted in a private room at the health facility. Data collection continued until data saturation was reached, data saturation was reached by the 9th or 10th interview depending on the County. Interviews were audio-recorded, transcribed verbatim, cleaned, and coded.

### Data analysis

Data was analyzed using NVivo™ Version 14 software. Thematic analysis steps involved: (1) thorough engagement with the transcripts through iterative readings to gain a deep understanding of the content; (2) assigning labels to identified codes and highlighting excerpts corresponding to each code; (3) formulating categories based on overarching trends observed in the data; (4) scrutinizing categories and merging or eliminating as required; (5) organizing data into themes and subthemes.

The team of investigators included six nurse midwives, an obstetrician, and a health systems expert. Trustworthiness was ensured by one investigator coding, categorizing the data, and identifying themes independently. The other investigators reviewed the themes and corresponding quotes. Interpretations were subjected to comparison and consensus among all the investigators. Direct quotes of participants were utilized to establish credibility. Transferability was ensured using a thick description of the methods employed in this study. Dependability was maintained through engagement of all the investigators in the analysis processes. Confirmability was established through clearly stating the steps taken in data collection and analysis for this study.

### Ethics

Ethical approval was obtained from the Kenyatta University Ethics and Review Committee (Ref No PKU/2563/1689) and the National Commission for Science, Technology & Innovation (Ref. No NACOSTI/P/22/19185), and County Research Boards. Informed consent was obtained from participants and confidentiality maintained.

Table 1.

**Table 1:** Sociodemographic Characteristics of Health Care Providers.

Variables	*n* (%)^a^
**Age**	
20–29	11(15)
20–39	37(49)
40 and above	26 (35)
**Gender**	
Male	29 (38)
Female	47 (62)
**Cadre**	
Nurse-Midwife	54 (71)
Clinical Officers	11(14)
Sonographer/radiographer	8(11)
Physicians	3(4)
**Area of deployment**	
Antenatal Care/Maternal Child Health	39(52)
Maternity	23(30)
Reproductive health	7(9)
Radiography	7(9)
**Facility level**	
Two	7(9)
Three	23(30)
Four	46 (61)
**Years of experience current facility/ position**	
<1year	7 (9)
1–9 years	57 (75)
10 and above	12 (15)
**Years of experience in the profession**	
1–9 years	36 (51)
10 and above	35(49)

a Totals across variables vary due to missing data.

### Findings

The majority (47/76) of the participants were female and mostly nurse/midwives (54/76). Most participants were aged between 20 and 39 years and worked in level four facilities with 1–9 years of experience. Five themes with subthemes emerged from the findings: (1) clinical decision-making, (2) quality of services, (3) O-POCUS training, (4) technology issues, and (5) O-POCUS sustainability. These are summarized in Table 2 with illustrative quotes.

**Table 2:** Sample themes and sub-themes.

Themes	Subthemes	Sample Quotes
**Clinical decision-making**	Confidence in Practice	“*I am sure of the decisions I am making, and when I am making a management plan for these patients, I am more confident in my approach having the POCUS here…*” (Clinical Officer-4).
	Augmentation of Physical Assessment	*“When you are palpating a client and you are not sure of the presentation or you are not getting the fetal heart, we use the POCUS ultrasound to confirm.”(Nurse/Midwide-20)*
	Interprofessional Collaboration	*“sometimes images may not be clear, we have been working together and if the nurses have a challenge, they come to me. They even come when they don't have clients in the ward, we never lack clients here in radiology”(Sonographer/Radiographer-7)*
**Quality of Services**	Increased Demand for ANC Services	*“The demand is high. Like every client who comes to the ANC wants ultrasound and to be done.” Nurse/Midwide-19)*
	Timely and Appropriate Referrals	*“It has helped us in better management of the pregnant mothers, when you get an abnormality, you refer the patient immediately. It is good for our facility since it has reduced maternal deaths.”(Sonographer/Radiographer-4)*
	Increased Workload	*“At times I am on duty alone and have so many clients. I am the one to conduct deliveries, prepare clients due for theatre, and take care of them post-delivery…It is a lot of work. So, workload is a challenge.” Nurse/Midwide-25)*
	Personnel and Resource Challenges	*“Staffing is an issue…we now do selective POCUS. We are not doing it routinely unless when you are not sure of the presentation.” Nurse/Midwide-18)*
		*“We have a challenge with paper towels and gel, because now we are using KY jell, we don't have the ultrasound gel.” (Clinical Officer-10).*
**O-POCUS training**	Perception of O-POCUS Training	*“One, the training material at that time was good. The trainers were good and even if you contact them over the phone, they respond….What can I say? I think I liked everything. I liked everything…” (Nurse/Midwide-12)*
	Proposed Areas of Improvement	*“The training was short and we needed to explore more. We needed more case scenarios, more abnormalities in that learning time we were there.” (Nurse/Midwide-22)*
**Technology issues**	Quality of Machines	*“You see this tablet is of very high quality it displays good images so sometimes our machine may not display as good so we use the tablet confirms something.” ”(Sonographer/Radiographer-3)*
	Technical Barriers	*“There is also an issue with updating the equipment. The machine updates itself sometimes when you are using it to perform.” (Clinical Officer-2)*
**O-POCUS Sustainability**	Training more HCPs	*“I think frequent Continuous Medical Education (CMEs) would go a long way*…*offer refresher courses for those who have been trained on POCUS… Even if it is just an online course.” (Physician-1)*
		*“On the job training should be offered to the others so that at least even when you go on leave, there will be continuity.” (Nurse/Midwide-5)*
		*“It is sustainable only if more are trained instead of relying on only one person in a facility. In that, when am not there, somebody else can do.” (Nurse/Midwide-22)*
	Mentorship and Support Supervision	*“We also require mentorship, and continuous supervision, remember since you trained us this is your first time that you are coming here.” (Nurse/Midwide-38)*
	Stakeholder Engagement	*“Yes, it would be better if the county government can help in the supply of the resources that we are lacking such as gel, towels, and maintenance of the ultrasound machines.” (Nurse/Midwide-14)*

**Theme 1: Clinical Decision-Making**

For the first theme, clinical decision-making, three subthemes emerged: (1) confidence in practice, (2) augmentation of physical assessment, and (3) interprofessional collaboration.

***Subtheme 1: Confidence in Practice***

Use of O-POCUS decreased fear, doubts, and uncertainties among healthcare providers when providing care. Participants felt that the confidence and the skills gained translated into appropriate and timely management decisions as seen in the following narratives:

*“You know we have a poor referral system and sometimes you are between a rock and a hard place… You cannot refer and at the same time decide to monitor labor or a mother, whose outcome you are uncertain. But because of the availability of O-POCUS, it gives us lots of confidence… we can now confirm and make appropriate decisions as opposed to before.” (Nurse/Midwife-83)*

*“O-POCUS is an advantage to the midwife because you get to be sure of what you're managing at that time without even sending the client to the radiology.” (Nurse/Midwife-70)*

*“I am sure of the decisions I am making, and when I am making a management plan for these patients, I am more confident in my approach having the O-POCUS here and doing it myself.” (CO-4)*

Some healthcare providers, however, reported that they had not achieved their desired level of confidence in identifying some conditions, *“I am not excellent because I still have challenges in locating the placenta*.” (Nurse/Midwife-6). Despite the challenges, other participants were optimistic that they would develop the desired level of confidence with more practice and experience, *“…am not yet there but with more practice I will perfect my skills.” (*Nurse/Midwife-33)

***Subtheme 2: Augmentation of Physical Assessment***

Healthcare providers recounted how O-POCUS played an important role in their clinical decision-making. Majority reported that O-POCUS provided a tool to confirm their suspicions when providing care to clients:

*“Instead of querying the presentation after palpation, you now confirm it with ultrasound.” (Nurse/Midwife-3)*

*“Before POCUS… you could use the fetoscope to auscultate the fetal heartbeat. At times you doubted, but O-POCUS, shows you how the fetal status is.” (Nurse/Midwife-7)*

*“O-POCUS confirms your suspicions that you had before referring a client for specialized care.” (Radiographer/ Sonographer-8)*

Rather than operating on assumptions, healthcare providers felt that they can rely on accurate data from O-POCUS, which improves the care decisions made. Some healthcare providers reported that O-POCUS had replaced some aspects of the physical examination:

*“O-POCUS has transformed how we perform physical examination…Now you can use your probe and auscultate fetal heart rate easily.” (Nurse/Midwife-3).*

*“since I started doing these scans’ I've not used the other fetoscope.” (CO-6)*

*“We do not rely on physical examinations we go directly to O-POCUS…and we have even forgotten to do a physical examination.” (Nurse/Midwife-46)*

***Subtheme 3: Interprofessional Collaboration***

The importance of interprofessional collaboration in improving healthcare providers clinical decision-making was reported. This collaboration informed decisions on referral of clients to other facilities or to other providers for advanced care and management:

*“…I refer patients to the sonographer. Maybe you want to confirm gestation, and you refer to the sonographer…Sometimes we normally send them to the sonographer to confirm…” (Nurse/Midwife-5)*

*“Once I encounter an abnormal finding I usually send the client to the sonographer for clarification. I refer for further clarification.” (CO-3)*

*“So, we have seen many referrals from ANC to the sonographer for confirmation, which is a positive thing…” (Physician-1)*

The above narratives reveal the crucial role that collaboration between different cadres of healthcare providers plays in improving diagnostic accuracy.

**Theme 2: Quality of Services**

Healthcare providers perceived that O-POCUS impacted the quality of services they offered. Four subthemes emerged (1) increased demand for ANC services, (2) timely and appropriate referrals, (3) increased workload, and (4) personnel and resource challenges.

***Subtheme 1: Increased Demand for ANC Services***

Healthcare providers reported that, due to O-POCUS, there was an increased demand for ANC services. They attributed the increased demand to the availability of O-POCUS. Most clients were demanding to get an ultrasound during their visit:

*“The demand is high. Every client who comes to the ANC wants ultrasound to be done.” (Nurse/Midwife-19)*

*“It has an impact because the mothers are increasing and most of them want to get an ultrasound.” (CO-10)*

***Subtheme 2: Timely and Appropriate Referrals***

Healthcare providers agreed that undergoing training on O-POCUS improved their clinical judgment especially in terms of timely referrals of clients for specialized care or confirmation of the diagnosis. The referrals were made to other providers within the same facility or to a facility providing higher level services. In most cases the timely decisions to refer clients had a positive impact on quality of care:

*“It has helped us in better management of the pregnant mothers, when you get an abnormality, you refer the patient immediately. It is good for our facility; it has reduced maternal deaths.” (Radiographer/Sonographer-4)*

*“O-POCUS has helped us in diagnosing risks and referring for comprehensive scan and further management to a higher level facility.” (Nurse/Midwife-89)*

*“We had a case of placenta previa, which was referred for specialized care since we don't have a theater here. A comprehensive ultrasound confirmed our diagnosis… a cesarean section was done successfully.” (Nurse/Midwife-48)*

*“… for example, when we get twin pregnancies, we are able to refer early… We do not even allow the mother to go into labor here, because we do not have a theatre… We just refer the mother when we get the twin pregnancy. So, it has really helped.” (Nurse/Midwife-44)*

Healthcare providers indicated that O-POCUS also helped in avoiding unnecessary referrals. The role of O-POCUS on reducing unnecessary referrals can be seen in the following narratives:

*“Fewer referrals. Long ago when we were not sure of the presentation, we would refer the mother but now when they come and you confirm the presentation is cephalic you are not worried.” (Nurse/Midwife-53)*

*“It has really helped me a lot and for instance, I've told you now when I want to see Doppler studies for the fetus I will not refer outside, I will just use the POCUS machine. (Radiographer/Sonographer-7)*

Overall, the positive impact of O-POCUS on quality of services is reflected in the timely decisions to refer followed by a confirmation of the diagnosis thereafter.

**Subtheme 3: Increased Workload**

Increased workload was reported as a barrier to integration of O-POCUS into routine practice. In some facilities, only one HCP was trained on O-POCUS making it difficult to perform it on every client. Clients attending ANC also increased due to the free O-POCUS service:

*“The high workload is because I am the only one trained on O-POCUS in the facility.”*

*(Nurse/Midwife 10)*

*“At times I am on duty alone and have so many clients. I am the one to conduct deliveries, prepare clients due for theatre, and take care of them post-delivery…It is a lot of work. So workload is a challenge.”(Nurse/Midwife 25)*

*“The main challenge is the workload****…****because I run this clinic alone.” (CO-5)*

**Subtheme 4: Personnel and Resource Challenges**

Most healthcare providers highlighted staff shortages as one of the organizational barriers to integration of O-POCUS into practice. In lower-level facilities, healthcare providers are expected to care for clients in various departments. Due to personnel shortages, O-POCUS is only performed for selected cases. Lack of essential supplies such as gel also hinders the implementation of O-POCUS. Together, these impacted their ability to offer high quality equitable services to all ANC clients.

*“Sometimes you are alone and you are running different departments. So, you cannot do O-POCUS on all the mothers…you pick those who are indicated to see if they have a problem.” (Nurse/Midwife-6)*

*“Staffing is an issue…we now do selective O-POCUS. We are not doing it routinely…” (Nurse/Midwife-18)*

*“We usually have a problem with the gel. Gel is the main requirement, most of the time is out of stock. And sometimes when you don't have it, there's no way you can use the equipment.” (Radiographer/sonographer-1)*

*“The only challenge that we have not been able to curb is availability of the towels to wipe out the gel from the patient.”(CO-5)*

*“… paper towels, we don't have so, I carry an extra tissue paper in my bag to wipe.” (Nurse/Midiwfe-89)*

**Theme 3: O-POCUS Training**

Participants shared different views regarding the training they received on O-POCUS. Two subthemes emerged under this theme: (1) perception of O-POCUS training and (2) proposed areas of improvement.

**Subtheme 1: Perception of O-POCUS Training**

Generally, healthcare providers who underwent the O-POCUS training had positive feedback about the program. Findings show that healthcare providers were especially impressed with the short, focused sessions that closely integrated theory and practical sessions. Healthcare providers appreciated the hands-on experience with real clients, an aspect that they described as beneficial in gaining practical skills. They also appreciated the comprehensive coverage of the content in addition to having skilled and knowledgeable trainers:

*“…the training materials were good, trainers were good, even if you contact them over the phone, they respond… I liked everything…” (Nurse/Midwife-12)*

*“I think what I liked most was the, the practical part of it, and it was the best training I've ever attended. Because it was learning theory for 30 min, then you go for practical's.” (Nurse/Midwife-06)*

*“Training facilities and demonstrations were nice…. they gave us ample time to do the practical's…” (Nurse/Midwife-27)*

*“The training was good, we had good instructors who were able to take us through not forgetting that us, as nurse/midwives we didn't know how to use the machine, we normally see the results without knowing what they are, but they made sure we came out knowing most of the things…” (Nurse/Midwife-52)*

Healthcare providers reported that the additional reference materials provided during the training was useful in enhancing their knowledge and skills:

*“We have the modules…. and they are of great importance…. In case you are missing something, you're able to refer to the modules and you are sorted.” (Nurse/Midwife-50)*

*“…the, iPad has notes, you keep on reading. Sometimes, at least when you are free, they have those notes… I read from the iPad, there are videos there that guide us…in case I'm forgetting something, I go back and read from there.” (Nurse/Midwife-49)*

**Subtheme 2: Proposed Areas for Improvement**

Healthcare providers suggested a longer period for training to enable them to get exposed to more practice and exposure to a broader variety of cases. Some healthcare providers believed that more time was needed to sharpen their skills further:

*“… training was short and we needed to explore more. We needed more case scenarios, more abnormalities in that learning time we were there.” (Nurse/Midwife-23)*

*“The training was inadequate. We feel the training could be a longer period and more technical people can be trained, then the program can continue…, the training we went was only one week… It was short time, and we did not explore many issues…” (Radiographer/Sonographer-1)*

Others proposed additional training to cover more topics beyond the five areas initially trained on:

*“…it was not adequate because I am not able to do a gestational age.” (Nurse/Midwife-20)*

*“Sometimes what challenges me is the question on gender of the baby by the mothers … you try to explain to her that this shows only how the baby is but when you go to another facility you'll be told about the gender …” (Nurse/Midwife-53)*

*“I feel like it could be important to learn how to calculate the gestation age because there are mothers who conceive while on family planning, they cannot tell you when they conceived because the last period was like four years ago.” (Nurse/Midwife-54)*

In summary, healthcare providers recognized the benefits of the O-POCUS training but highlighted areas where additional skills, knowledge, and support would enhance their ability to provide comprehensive care to pregnant women. They emphasized the importance of addressing these aspects to improve the effectiveness of the O-POCUS program.

**Theme 4: Technology Issues**

Two subthemes emerged from the fourth theme of technology issues: (1) quality of machines, and (2) technical barriers.

**Sub-theme 1: Quality of Machines**

Healthcare providers indicated that the quality of O-POCUS devices made their work easier, given that they are portable and provide clearer images as compared to standard ultrasound machines. Furthermore, they outlined that it was easy to show mothers the status of their fetus on the tablet screen due to its portability:

*“I have interacted with my colleagues, the radiographers here. They know how to use that tablet; they can do any exam using it. You see this tablet is of very high quality it displays good images so sometimes our machine may not display as good, so we use the tablet confirms something.” (Radiographer/Sonographer-3)*

*“It has made our work easier, the machine is portable, you can carry it everywhere… it is very beneficial to us.” (Radiographer/Sonographer-4)*

*“O-POCUS is so flexible that you can show the mother the way the baby is.” (Nurse/Midwife-69)*

**Sub-Theme 2: Technical Barriers**

Healthcare providers identified technical barriers that hinder the integration of O-POCUS into practice. Technical barriers such as unreliable power supply, inadequate internet access which was required to update the machine and upload images, and ultrasound machine failures were reported. Unreliable power supply interrupted the continous provision of O-POCUS services:

*“Sometimes power outage, being in a remote area, you find that there's no power, so charging the gadgets becomes a problem.” (Nurse/Midwife-11)*

*“sometimes you can go 3–4 days without electricity. That means the gadget will not function because of lack of power.” (Nurse/Midwife-10)*

*“We were not using O-POCUS because of internet challenges. This part is very remote…the issue of the internet is a huge challenge.” (Nurse/Midwife-84)*

*“Sometimes the machine does not connect to the network. This inconveniences the provision of ultrasound services.” (Nurse/Midwife-13)*

Other challenges such as tablets locking themselves or becoming unresponsive, overheating of the probe, interruption by untimed automatic updates, and shutting down in the middle of an assessment were identified.

*“We have experienced the challenge of the iPad locking itself.” (Nurse/Midwife-07)*

*“Sometimes the probe overheats…and, the charger might not function properly leading to the machine being not fully charged. Clients have to wait for long as you charge since the process takes time before being fully charged.” (CO-1)*

*“There is also an issue with updating the tablet. The machine updates itself sometimes when you are using it to perform the scans.” (CO-2)*

**Theme 5: O-POCUS Sustainability**

Three subthemes related to sustainability of O-POCUS practice were reported: (1) training more Healthcare providers, (2) mentorship and support supervision, and (3) stakeholder engagement.

***Subtheme 1: Training more Healthcare providers***

Most of the study participants recommended that more healthcare providers should be trained on O-POCUS to enhance its full implementation and ensure its sustainability. They perceived that training more healthcare providers would address the high workload experienced by the few O-POCUS-trained healthcare providers. Training more staff on O-POCUS would enhance the quality and consistent availability services offered in their respective counties:

*“We need more training of more staff because we have done on the job training (OJT) for staff, but you see there are still gaps… I believe if we could get more trainings, maybe even at the county level by you guys it could be better… The way we were trained and the way we are mentoring them there are still gaps. Because you find you will mentor someone, but they still want you to do it for them. They don't have the confidence. And I think there are some details that we cannot sit down teach them the way we were taught. So, they could really benefit from more training. (CO-4)*

*“Maybe we train more than two staff in maternity. I am the only trained staff in maternity… the workload is high. I am even called when off-duty to come and do O-POCUS when the sonographers are not there.” (Nurse/Midwife-22)*

*“.. .if the number of people who know O-POCUS increases that means at every shift at any time the mother can benefit from O-POCUS … there were few who were trained. So if everyone was trained it would be easier and improve the outcome for the mothers.”(Physician-2)*

Most participants agreed that the provision of on-the-job training (OJT) was important for the sustainability of O-POCUS. By training others in their facilities on O-POCUS use, they asserted that OJT would improve continuous service delivery for when trained personnel are not available. Participants also felt that inter-professional OJT to other provider cadres could enhance POCUS implementation, resulting in improved service delivery.

*“Here we are doing OJT to some of the nurses, but if the scope of training can be widened, to include more clinical officers and medical officers, I think it can have an enormous impact on service delivery. So, you strengthen and train more staff here in Level Four hospitals and other peripheral facilities.” (Nurse/Midwife-50)*

*“In the future, we should offer OJT to all the nurses and any HCP in the hospital especially the clinicians because not all the clinicians have been exposed to O-POCUS. The best thing is to make sure everybody in the hospital knows how to use a O-POCUS.” (Nurse/Midwife-52)*

***Subtheme 2: Mentorship and Support Supervision***

Most participants agreed that continuing mentorship should be provided to healthcare providers for sustainability and quality assurance. They asserted that mentorship would help them improve their skills and address challenges they encounter in implementing O-POCUS.

*“We also require mentorship, and continuous supervision.” (Nurse/Midiwfe-38)*

*“We just want the mentorship and more training in terms of how much more, what more we can do with O-POCUS. (CO-3)*

*“Mentorships will ensure that skills are not lost.” (Physician-1)*

*“…for O-POCUS we have not received enough support on its implementation…I think we could provide better services if we would get mentorship and the technical support for the challenges we encounter with O-POCUS.” (Nurse/Midiwfe-33)*

*“Maybe more training, supervision, and support, we can also learn how to estimate the gestation…”(Nurse/Midwife-51)*

Refresher courses and continuing education opportunities were also recommended to ensure healthcare providers remain updated on current practices and sharpen their skills on O-POCUS.

*“Sometimes, we need regular updates.” (Nurse/Midwife-1)*

*“We need more trainings and refreshment of the course. (CO-2)*

*“I think frequent Continuous Medical Education (CMEs) would go a long way…offer refresher courses for those who have been trained on O-POCUS… Even if it is just an online course.”(Physician-1)*

***Subtheme 3: Stakeholder Engagement***

Government's role in facilitation of O-POCUS sustainability was evident in the narratives of the participants. They agreed that the government should be proactively involved in supporting integration of O-POCUS into routine health care services. They identified various ways the government could provide support, including procurement and maintenance of ultrasound machines, program management support, provision of essential supplies, refresher training and training of more staff.

*“County governments might be involved more on issues such as training and supply of the required commodities.” (CO-7)*

*“Yes, it would be better if the county government can help in the supply of the resources that we are lacking such as gel, towels, and maintenance of the ultrasound machines.” (Nurse/Midwife-14)*

Integration of O-POCUS services into the existing government health policies was also identified as a key component toward sustainability, including incentives to hard working and excelling staff.

*“… the county should reward those who are trained to offer O-POCUS services to clients to motivate them.” (CO-8)*

*“…routine use of O-POCUS might be considered to be part of government or organizational policies. You can also come up with awards for the people who are doing better in the delivery of O-POCUS services in different facilities.” (Nurse/midwife-54)*

Overall, the narratives of the participants revealed that O-POCUS sustainability is dependent on training more healthcare providers, mentorship and continuing education for trained providers, receiving incentives, and organizational and government support.

## Discussion

Our results showed healthcare providers perspectives in using O-POCUS and the role it plays in clinical-decision making and quality of services provided. Although some technological and implementation issues were raised, proposals for sustainability were also identified. Overall, these findings are consistent with Suttels et al. (2023) and Wanjiku et al. (2024) who found that O-POCUS offers a solution due to its ease-of-use, portability, cost-effectiveness, and diagnostic accuracy, thereby improving healthcare delivery in sub-Saharan Africa.

In LMIC'S, limited access to radiology services can exacerbate health disparities and make it difficult to comply with WHO guidelines (Bidner et al., 2023; Doran et al., 2022; Suttels et al., 2023). This study further underpins the importance of expanding strategies to bringing ultrasound services closer to communities. Specifically, participants who primarily worked in lower-level facilities demonstrated positive attitudes towards O-POCUS, expressing confidence in its utilization. These findings echo previous research by Baloescu et al. (2022), Viner et al. (2023), Kim et al. (2021), and Wanjiku et al. (2024), highlighting O-POCUS's potential to reduce diagnostic uncertainty and provide patient-centered care in LMICs.

Our findings show that O-POCUS plays a significant role in clinical decision-making, consistent with many studies (Andersen et al., 2020; Wachira et al., 2023; Haldeman et al., 2022; Kumar et al., 2023; Wanjiku et al., 2024) that report the same conclusion. Our study, extends these findings with evidence of interprofessional collaboration. As this study included a diverse cadre of healthcare providers in different levels of facilities, our findings demonstrate how O-POCUS task sharing was perceived among nurse/midwives, clinical officers and sonographers/radiographers. Of note, respondents felt that O-POCUS positively impacted the timeliness and appropriateness of referrals.

Our findings reinforce that access to ultrasound increased the demand for ANC services among pregnant mothers. Other studies (Doig et al., 2019; Knights et al., 2023) have shown that O-POCUS increased the uptake of ANC services, which improved maternal and neonatal outcomes because of the high number of antenatal interventions provided. However, with increased demand of O-POCUS, healthcare providers described increased workload as a potential barrier to implementation, which was exacerbated by workforce shortages. Despite this challenge, participants offered recommendations for increasing the number of healthcare providers who could administer O-POCUS through OJT.

Healthcare providers voiced suggestions for improvement, including the extension of training periods and expansion of curriculum coverage through additional topics, aiming to enhance the program's efficacy. Moreover, the establishment of training centers (Kim et al., 2021) and the integration of ultrasound training into medical and nursing schools (Wachira et al., 2023; Lukhele et al., 2023) are deemed crucial for positively impacting patient care in the future and ensuring the sustainability.

Healthcare providers appreciated the quality and portability of O-POCUS devices. However, barriers such as unreliable power supply, inconsistent availability of consumables, and equipment malfunctions were identified, consistent with previous studies (Kim et al., 2021; Suttels et al., 2023; Stone et al., 2021; Ginsburg et al., 2023). These challenges underscore the need to strengthen underlying health system factors.

The study also found that the sustainability of O-POCUS relies on several key factors, including the expansion of health care provider training, the establishment of mentorship programs, and active engagement of stakeholders. In addition, government involvement was deemed crucial in providing essential support, allocating resources, and developing policy frameworks to ensure quality standards are met for the long-term success of O-POCUS initiatives. This observation is corroborated by existing literature, which underscores the importance of various sustainability strategies such as capacity building, formulation of policy guidelines, continuous training with onsite supervision, professional development, and mentorship programs for quality assurance (Viner et al. 2023; Mbakaya et al., 2020). Clear regulatory guidelines and specialist supervision are recommended to ensure quality assurance and to facilitate the ongoing utilization of O-POCUS in obstetrics and midwifery (Kumar et al., 2023).

### Strengths and limitations

Participants represent views of trained providers across different levels of facility which is a strength of the study. However, we were unable to match participants and facility level and are thus unable to distinguish the views from those in lower or higher level facilities. Additionally, factors such as desirability bias may have influenced results as healthcare providers shared their experiences with O-POCUS. This limitation should be acknowledged when interpreting and transferring the study's findings to other settings.

## Conclusion

This study revealed that it is possible to train healthcare providers on O-POCUS across cadres on a large scale in order to enhance clinical decision-making and improve quality of services, and that healthcare providers in low-resource settings have an overall positive view of O-POCUS when incorporated into their clinical care. Addressing training, technological, and sustainability challenges is imperative for maximizing its impact in LMICs.

## CRediT authorship contribution statement

**Lister N. Onsongo:** Writing – review & editing, Writing – original draft, Visualization, Validation, Supervision, Methodology, Investigation, Funding acquisition, Formal analysis, Conceptualization. **Sarah C. Bett:** Writing – review & editing, Writing – original draft, Validation, Methodology, Investigation, Funding acquisition. **Grace W. Gachuiri:** Writing – review & editing, Writing – original draft, Validation, Investigation. **Stephen N. Njuguna:** Writing – review & editing, Writing – original draft, Validation. **Jacob W. Masika:** Writing – original draft, Validation, Investigation, Funding acquisition. **George O. Otieno:** Writing – review & editing, Writing – original draft, Investigation, Funding acquisition. **Anthony K. Wanyoro:** Writing – review & editing, Writing – original draft, Validation, Investigation, Funding acquisition. **Matthew S. Haldeman:** Writing – review & editing. **Dilys Walker:** Writing – review & editing, Writing – original draft, Validation, Methodology. **Nicole Santos:** Writing – review & editing, Writing – original draft, Validation, Methodology, Conceptualization. **Grace K. Githemo:** Writing – review & editing, Writing – original draft, Validation, Investigation, Funding acquisition.

## Declaration of competing interest

The authors declare that they have no known competing financial interests or personal relationships that could have appeared to influence the work reported in this paper.

## Appendix Supplementary materials

Image, application 1

Image, application 2

Image, application 3

Image, application 4
