# Supplementary material for: Perspectives of health care providers on obstetric point-of-care ultrasound in lower-level health facilities in Kenya
Source: Midwifery. 2025 Jan;140:104196. doi: 10.1016/j.midw.2024.104196 (PMC11619753; doi:10.1016/j.midw.2024.104196)
Supplement: Supplementary file 1 [file mmc1.docx]

# **QUALITATIVE DATA COLLECTION TOOLS**

# **HEALTH CARE PROVIDERS QUALITATIVE DATA COLLECTION TOOL**

**Introduction:**

Thank you for agreeing to take part in this interview. Before we begin, I would like to clarify some information with you. With your permission, the interview will be audio recorded. There are no ‘right’ or ‘wrong’ answers to the questions I will ask you. I am interested in your opinions and experiences of the ultrasound examinations you performed. I will take out the names of anyone you mention during the interview to protect their identity. Your participation will not affect your employment in any way. If there are any questions that you do not wish to answer, please say so and we will move to the next question. If you wish to pause or stop the interview at any time, again, please let me know. Are there any questions that you would like to ask me about the interview before we begin?

Are you happy to proceed?

Are you okay with the interview to be recorded?

**Biographical data:**

1. Level of Facility--------------
2. Area of deployment___________ (ANC, labor ward)
3. Gender-----------------
4. Age-------------------
5. Profession/Cadre______________ (nurse, CO, doctor)
6. Position……………
7. Years of experience____________
8. No of months since being deployed in your current department—---------
9. When were you trained in POCUS? *(specific dates)

**REACH**

- - - 1. In your opinion, how often have you been performing POCUS since the time you were trained?

*Probe: reasons/explanation given for the frequency, probe for challenges etc*

How has the trend in attendance of ANC been impacted by the introduction of POCUS? *Probes: Have you seen more mothers? The same number of mothers? Please explain why you think this is so.*

1. Tell me about the demand for the US in the Labor wards.

*Probes: Do all women get scanned? If not, how do you select women to scan?*

**EFFECTIVENESS**

1. Have you ever encountered an abnormal finding when performing a POCUS? Tell me about it? What did you do? How did the mother respond? Whenever you encounter a finding that is out of the ordinary/expected what actions do you take (abnormal finding POCUS), what do you do?

*Probes: referral to radiology for second opinion, referral out of the facility) to ensure continuity of care, other; documentation of findings and where*

1. In your opinion and based on your experience with the use of POCUS, how has it impacted the type of care you provide?
2. *Probes: perceived improvements in diagnosis of complications, clinical decision making and/or referrals; increased knowledge/confidence in POCUS; neglect of other services due to workload.* How has provision of ANC/labor ward services been impacted?
3. How has POCUS changed how you conduct a physical examination (abdominal palpation & auscultation)?

Probes: perceived benefit of POCUS over standard of care (e.g., accuracy of diagnosis)

**ADOPTION**

1. *In your opinion*, to what extent is POCUS being used in your daily practice to manage women antenatally and/or in labor?

Probes: Frequency of use & indication (e.g., fetal presentation); reasons for limited uptake; preferred setting for use (ANC or labor) What are some of the things or issues that prevent *(or would prevent)* you from incorporating POCUS in the management of clients in your practice? During ANC care? During labor? Are they the same factors or different depending on ANC/Labor? Why? What factors impact availability when you want to use the US?

*Probes:*

- *Individual: confidence, skills, workload*
- *System: lack of gel, paper towels, workload, bundles electricity etc.?*

**IMPLEMENTATION**

1. What are the most common factors that informs your use of POCUS most of the time?

*Probe: Are there specific situations when you tend to use POCUS more than others*

1. In your opinion, how does implementation of POCUS affect your workflow?

Probes: more or less time with patient, efficiency in delivering care

1. *In your opinion, what resources were put in place that enhance use of ultrasound in your facility? What resource impeded implementation?*

*Probes: mentorship, access to machine, availability of supplies*

**MAINTENANCE**

1. In your opinion, are there structures/strategies that should/have been put in place to enhance the use of POCUS in your day to day practice?

Probes: *Champions/mentorship/supervision….CPD’s, workshops. Technical support, supplies, deployment*

In your opinion, how do you foresee the sustainability of POCUS will be in the facility that you are working in? What would be needed to make it sustainable? In ANC, in Labour ward? What makes you believe it is reasonable or unreasonable that all women will get a POCUS before 24 weeks? In Labour?

*Probes: Sustainability resources (how it affects facility, time, workload, financial streams), role of sonography department staff, task shifting, policy/guidelines/SOPs documents availability, internal initiatives*

**QUESTIONS ABOUT TRAINING**

1. What do you think about the POCUS training which you underwent? *Was it helpful/not helpful?*

Probe: *What did you like most about the training and what did you not like? Please explain what do you propose can be done to enhance the training?*

1. How do you assess your skills and experience in the provision of POCUS scanning services?
2. What kind of feedback have you gotten on your PCOUS skills since the training? Is this feedback useful? What kind of feedback would be more useful.
3. Have you become confident in using POCUS for assessments that you received training?

*Probes: do you see any improvement as you continue performing? Do you see like your performance has improved over time as you continue to perform?*

1. Have you explored other assessments not specifically taught during the training?

*Probes (e.g., Gestational Age) If so, what? Why? Tell me about how easy it was to learn this skill.*

1. What further training or support would you need to improve your practice?

*Probes: refresher training, mentors,*

1. After your POCUS training, how have you shared the knowledge and skills with other providers?

*Probe: training others, receptiveness, & interest to learn*

**Any additional comment overall that we may not have discussed?**

**Closing Comments**

Thank you so much for taking your time to participate in this interview today.

# 
