# Supplementary material for: Perspectives of health care providers on obstetric point-of-care ultrasound in lower-level health facilities in Kenya
Source: Midwifery. 2025 Jan;140:104196. doi: 10.1016/j.midw.2024.104196 (PMC11619753; doi:10.1016/j.midw.2024.104196)
Supplement: Supplementary file 2 [file mmc2.docx]

**Intervention**

The intervention involved training 514 HCPs (nurse/midwives, clinical officers (CO), and radiographer/sonographers) on O-POCUS working in maternity, ANC, and radiology departments in eight counties. Cohorts of approximately 50 HCPs underwent a 5-day training by Global Ultrasound Institute. Each trainee was provided with a Butterfly iQ+^TM^ ultrasound probe and an iPad by Butterfly Network. Recruitment of trainees was conducted by Kenyatta University. The training took place for a period of 10 weeks from September to December 2022. Topics covered included identification of fetal presentation, multiple gestation, fetal heart rate, placental position, and amniotic fluid assessment using the single deepest pocket method. Full details of the training have been published elsewhere (Wachira et.al., 2023).
