# Supplementary material for: Perspectives of health care providers on obstetric point-of-care ultrasound in lower-level health facilities in Kenya
Source: Midwifery. 2025 Jan;140:104196. doi: 10.1016/j.midw.2024.104196 (PMC11619753; doi:10.1016/j.midw.2024.104196)
Supplement: Supplementary file 3 [file mmc3.docx]

**Overview of the Health Care System in Kenya**

The health care system in Kenya is made up of three main sectors: government (42%), non-governmental organizations (NGOs) (15%), and private entities (43%). While all three sectors offer primarily curative services, there is limited focus on preventive health care. Most health care facilities—including hospitals, health centres, and dispensaries—are government-run, though the private sector plays a significant role by operating hospitals, clinics, and nursing homes across the country.

Government-owned facilities provide services through a structured network of health facilities categorized into six levels (Table 1).

**Table 1: Classification of Health Facilities in Kenya**

| **Level** | **Responsibility** | **Description** |
| --- | --- | --- |
| 1 | Community level | Offers preventive services through health education and case identification, with referrals to higher levels. Staffed by volunteer community health providers. |
| 2 | Dispensary or clinic | Provides both curative and preventive services, including basic outpatient care, laboratory services, maternity care, minor surgeries, and limited inpatient facilities |
| 3 | Health centre | Provides a wider range of outpatient services and preventive care, often serving as a referral point for Level 2 facilities. Have maternity facilities. |
| 4 | Sub-county hospital (Primary hospital) | Offers health care services within a specific geographical area, formerly at the district level. Acts as a primary hospital for the sub-county. They have a maternity and theatre facilities. |
| 5 | County hospital (Secondary hospital) | Provides specialized care, including accident and emergency services, surgery, medicine, obstetrics and gynaecology, paediatrics, psychiatry, intensive care, and more, at the county level. |
| 6 | National hospital (Tertiary hospital) | Functions as a referral centre, responsible for policy formulation, capacity building, research, and serving as internship or apprenticeship hubs for medical professionals. |


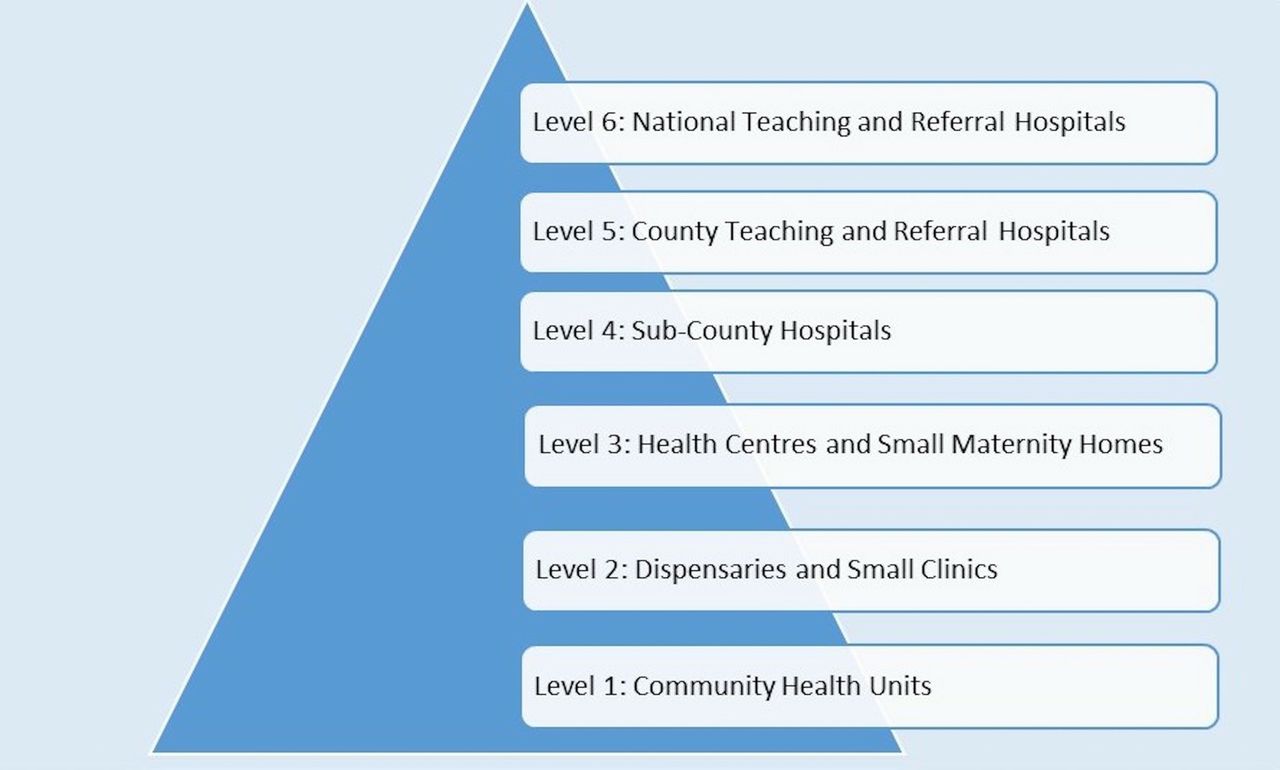


Source: <https://www.paukwa.or.ke/story-series/kehospitals/kenyas-healthcare-tier-system-explained/> retrieved 16/06/2024
