# Supplementary material for: Perspectives of health care providers on obstetric point-of-care ultrasound in lower-level health facilities in Kenya
Source: Midwifery. 2025 Jan;140:104196. doi: 10.1016/j.midw.2024.104196 (PMC11619753; doi:10.1016/j.midw.2024.104196)
Supplement: Supplementary file 4 [file mmc4.docx]

During the preparation of this work the authors used ChatGPT to edit an early version of the manuscript. After using the service for initial editing the authors proceeded with further revisions and edits individually. The authors take full responsibility for the content of the publication.
